# Subsurface Endospore-Forming Bacteria Possess Bio-Sealant Properties

**DOI:** 10.1038/s41598-018-24730-3

**Published:** 2018-04-24

**Authors:** Sreenivasulu Basha, Lakshman Kumar Lingamgunta, Jayakumar Kannali, Swarna Kumari Gajula, Ramesh Bandikari, Sreenivasulu Dasari, Veena Dalavai, Paramageetham Chinthala, Prasada Babu Gundala, Peera Kutagolla, Vinodh Kumar Balaji

**Affiliations:** 1 Department of Microbiology, Sri Venkateswara University, Tirupati, 517502 Andhra Pradesh India; 2 Department of Biochemistry, Sri Venkateswara University, Tirupati, 517502 Andhra Pradesh India; 3 College of Life Science and Technology, State key Laboratory of Agricultural Microbiology, Huazhong Agricultural University, Wuhan, 430070 China; 4 Department of Zoology, Sri Venkateswara University, Tirupati, 517502 Andhra Pradesh India; 5 Department of Civil Engineering, Siddharth Institute of Engineering & Technology, Puttur, 517583 India

## Abstract

Concrete is a strong and fairly inexpensive building substance, but has several disadvantages like cracking that allows corrosion, thus reducing its lifespan. To mitigate these complications, long-lasting microbial self-healing cement is an alternative that is eco-friendly and also actively repairs cracks. The present paper describes the detailed experimental investigation on compressive strength of cement mortars, mixed with six alkaliphilic bacteria, isolated from subsurface mica mines of high alkalinity. The experiments showed that the addition of alkaliphilic isolates at different cell concentrations (10^4^ and 10^6^ cells/ml) enhanced the compressive strength of cement mortar, because the rapid growth of bacteria at high alkalinity precipitates calcite crystals that lead to filling of pores and densifying the concrete mix. Thus, *Bacillus subtilis* (SVUNM4) showed the highest compressive strength (28.61%) of cement mortar at 10^4^ cells/ml compared to those of other five alkaliphilic isolates (*Brevibacillus* sp., SVUNM15-22.1%; *P. dendritiformis*, SVUNM11-19.9%; *B. methylotrophicus*, SVUNM9-16%; *B. licheniformis*, SVUNM14-12.7% and *S. maltophilia*, SVUNM13-9.6%) and controlled cement mortar as well. This method resulted in the filling of cracks in concrete with calcite (CaCO_3_), which was observed by scanning electron microscopy (SEM). Our results showed that the alkaliphilic bacterial isolates used in the study are effective in self-healing and repair of concrete cracks.

## Introduction

Microorganisms are the predominant life forms on all subsurfaces and are found wherever sufficient space, nutrients and water are available. Subsurface microbial populations are influenced by environmental factors like pH, temperature, hydrostatic pressure and dissolved salts. These microorganisms depend on several mechanisms to survive high pressure and temperature, low energy and nutrients, extreme acidity, alkalinity, metal toxicity, and radioactivity^1^. Previous studies have shown that microbial communities present in extreme ecosystems not only adapt to the above-mentioned conditions, but also change their environments in accordance with their lifestyle. Various useful functions and advanced technological applications have been identified by a comprehensive study of microorganisms in unusual environments, such as deep underground. Microbes also play key roles in mineral growth and rock weathering, formation of ore deposits, mobilization of metals, isotope fractionation, generation of porosity in deep surface and emergence of aerobic biosphere^2^. It is essential to perform in-depth research on microbial diversity to fill the gaps in performance, integration and evaluation of geochemical data.

In recent decades, microorganisms that grow under various physiological conditions have been derived from deep surfaces important in the sedimentary environment^3–6^. The imperfection of deep surface samples has been decreased with the latest sampling methods. Microorganisms retrieved from a deep subsurface core may allude to indigenous populations. The microorganisms recovered from deep surfaces are diverse and differ from the inhabitants of the surface^7,8^. Extensive analysis has demonstrated that a wide range of microorganisms are recoverable from the deep subsurface, and their actions may profoundly stimulate the geochemistry of subsurface environments. The subsurface environment and microorganisms have been subjected to geochemical analysis to determine whether they facilitate certain reactions that are thermodynamically favourable.

The subsurface biosphere has become an advanced area of research, both geologically and biologically. Thus, research requires input from taxonomy as well as quantitative multidisciplinary *in situ* experiments. According to current statistics, microorganismal diversity is increasing. However, 99% of microorganisms have not yet been cultivated. Therefore, it is a significant issue to link microbial diversity with the physiological functions and bio-geochemical roles of microorganisms.

Mortar concrete is one of the most important components in the construction industry because it is less expensive and more convenient than other materials. However, the disadvantage of concrete is that it cracks under constant loading due to environmental conditions, ultimately reducing the lifespan of a structure that is built with these materials^9,10^. The main concerns raised in most studies revolve around durability and strength^11–17^. In fact, these disadvantages take effect much earlier than the actual lifespan of the building construction. Synthetic substances such as epoxies are used as preventive materials to avoid cracks in building structures. However, they are expensive, not aesthetically pleasing and require constant maintenance. Bacteria serve as geochemical agents to raise the dispersion, fractionation, and strength of resources in the biosphere. Microbial mineral precipitation occurs during the metabolic activities of microorganisms. Based on the theory of bio-mineralization, a method was developed to create bio-concrete/biomortar material via the enrichment culture of alkaliphilic bacteria in concrete. The most important benefits of bio-concrete are the increased compressive strength and self-healing ability^18^.

The development of concrete technology considers the enhancement of its strength and durability using pollution-free and natural procedures while also addressing the design stage itself. The addition of *Bacillus subtilis* at a cell concentration of 10^6^ cells/ml to recycled concrete aggregates resulted in a 20% increase in compressive strength. The effects of calcium carbonate extend recycled concrete aggregate properties of the concrete^19^. Several studies applied microbial concrete, using various microorganisms, to influence the compressive strength of mortar and concrete^20–26^. The present study aims to develop an eco-friendly bio-concrete material via the enrichment culture of alkaliphilic isolates to increase the strength of concrete structures.

## Results

### Isolation and molecular identification of isolates

Six selected aerobic endospore-forming isolates were detached by means of cultivation-based analysis and labelled SVUNM4, SVUNM9, SVUNM11, SVUNM13, SVUNM14, and SVUNM15. The SVUNM4 isolate was a small rod-shaped bacterium; according to its 16S rRNA gene sequence homology, it was identified as a *Bacillus* sp. closely related to *Bacillus subtilis* sub sp. (GQ375226.1), with 100% homology. The SVUNM9 isolate was a gram-positive rod, which showed 99% identity with *B. methylotrophicus* (HQ831404). The SVUNM11 isolate was a gram-positive rod, which shared 100% identity with *Paenibacillus dendritiformis* strain p4111 (HM071942.1). The SVUNM13 isolate was a gram-positive rod identified as *Stenotrophomonas* sp., which is the nearest homologue to *Stenotrophomonas maltophilia* (JN644502.1), with 0.98% identity. The SVUNM14 isolate was a gram-positive rod. The 16S rRNA gene sequence homology identified it as a *Bacillus* sp., closely related to *B. licheniformis* (MM006903.1) with 99% identity. The SVUNM15 isolate was a gram-positive rod identified as *Brevibacillus* sp. N3, which is a close homologue of *Brevibacillus* sp. N2 (Gen.HQ231210.1). The molecular identification results for these six aerobic endospore-forming isolates are summarized in the Supplementary Information (Tables S1–S6 and Figures S1–S6).

### Morphological and biochemical characterization

All six isolates were gram-positive rods of varying sizes and tended to form small or long chains or remain as discrete entities. The isolate SVUNM4 was a pin head, medium to large in size, purple in colour, and circular in form, with an entire margin and convex elevation. The cultural characteristics of isolate SVUNM9 include small size, white colour, an irregular form, an entire margin and a raised elevation. The isolate SVUNM11 exhibited the following cultural characteristics: the colonies were large in size, cream in colour, and irregular in form, with a filamentous margin and flat elevation. The isolate SVUNM13 possessed the following cultural characteristics: small, white colonies with an irregular form and an entire margin showing flat elevation. The cultural characteristics of SVUNM14 were as follows: small-sized colonies with a light brown colour in a rhizoid form, with a lobate margin and flat elevation. Similarly, the isolate SVUNM15 exhibited light brown- to red-coloured colonies with a small size and circular form. All the isolates were spore-forming bacteria from mica, and a capsule was absent in all the isolates. Biochemical characterization of the isolates, including tests of assimilation and carbohydrate metabolism, were performed. All the isolates were positive for the Voges-Proskauer reaction, citrate utilization, gelatin liquefaction, starch hydrolysis and nitrate reduction (Table 1).

**Table 1:** Biochemical characteristics of endospore forming bacterial isolates.

S. No	Characters	SVUNM4	SVUNM9	SVUNM11	SVUNM13	SVUNM14	SVUNM15
1	SEM shape	rods	rods	rods	Rods	rods	Rods
2	Gram’s	−	+	+	+	+	+
	staining						
3	Spore staining	+	+	+, endospore	+	+	+
4	O/F test	Fermentative	Oxidative	Fermentative	None	+	Fermentative
5	Triple-sugar	K/A	K/A black	K/A Gas+H_2_S	K/A+H_2_S	K/A+H_2_S	A/A+H_2_S
	Iron test		precipitate		black	black	black
6	Catalase	−	+H_2_S	+	precipitate	precipitate	precipitate
			+		+	+	−
7	Indole	+	−	−	−	−	+
	production						
8	Methy red	−	+	+	−	+	−
9	Voges	+	+	+	+	+	+
	Proskaur						
	reaction						
10	Citrate	+	+	+	−	+	+
	Utilization						
11	Urease	+	+	+	+	+	+
	activity						
12	Starch	+	+	+	+	+	+
	hydrolysis						
13	Gelatin	+	+	+	+	+	+
	Liquifaction						
14	D- glucose –	+	+	+	+	+	+
	Acid						
	D- glucose –	−	−	−	−	−	−
	Gas						
	Lactose Acid	−	−	−	−	−	−
	Lactose Gas	+	+	+	+	−	+
	Sucrose Acid	+	+	+	−	+	−
	Sucrose Gas	−	−	−	−	−	−
15	Nitrate	+	+	+	+	+	+
	reduction						
16	H_2_S	+	+	+	+	+	+
	production						

Negative results (−), Positive results (+).

The SVUNM4 isolate is a fermentative microbe able to ferment glucose only; is negative for catalase and methyl red; is positive for indole and Voges-Proskauer; and is able to utilize citrate, starch, gelatin, sucrose, lactose, and glucose. The SVUNM9 isolate is oxidative-positive for catalase, methyl red, the Voges-Proskauer reaction, citrate utilization, starch hydrolysis, gelatin liquefaction and nitrate reduction and is negative for indole production. SVUNM9 has the ability to utilize glucose, sucrose, and lactose. The SVUNM11 isolate is fermentative; positive for catalase production, the methyl red test, the Voges-Proskauer test, citrate utilization, starch hydrolysis, gelatin liquefaction, and nitrate reduction; and is able to utilize glucose, lactose, and sucrose. The isolate SVUNM13 is negative for the oxidative-fermentative test, indole production, methyl red, and citrate utilization and positive for nitrate reduction, the Voges-Proskauer reaction, citrate utilization, starch hydrolysis, gelatin liquefaction and nitrate reduction. SVUNM13 is negative for the ability to utilize glucose, sucrose, and lactose. The SVUNM14 isolate is fermentative; positive for catalase production, methyl red, the Voges-Proskauer test, citrate utilization, starch hydrolysis, gelatin liquefaction, and nitrate reduction; is negative for indole; and is able to utilize glucose, lactose, and sucrose. The SVUNM15 isolate is fermentative; positive for indole, Voges-Proskauer, citrate, and nitrate reduction; and is able to hydrolyse gelatin and starch. SVUNM15 is negative for the methyl red and catalase tests and is able to utilize glucose and lactose.

### Growth studies of bacterial isolates

**Figure 1:**
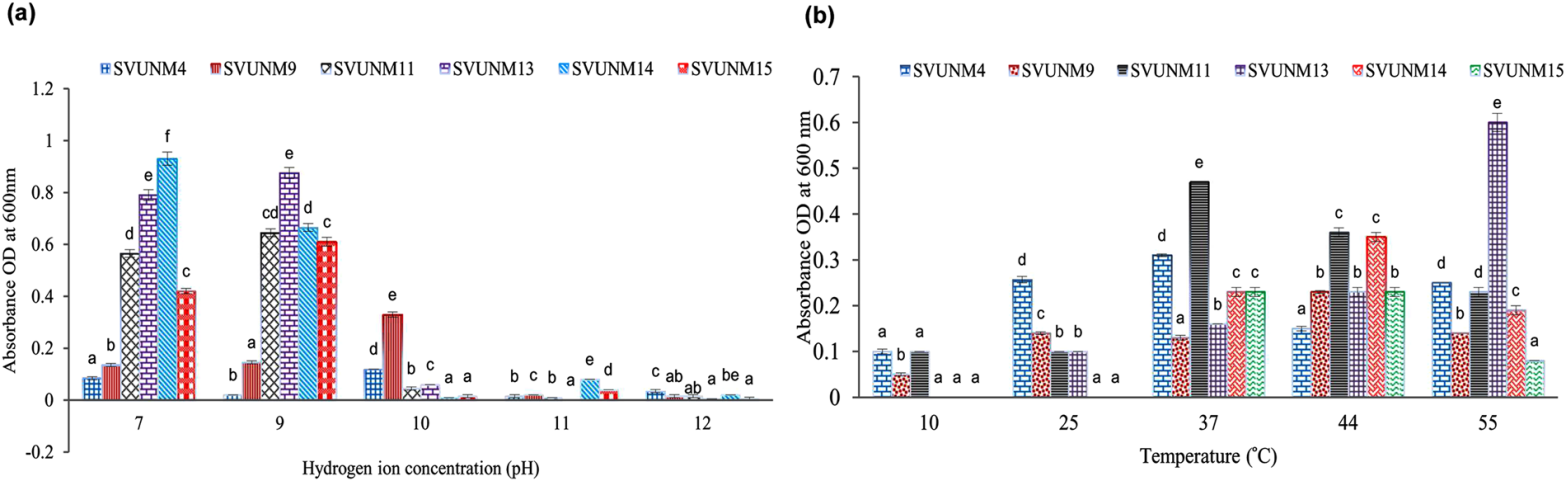
Effects of pH and temperature on the growth of alkaliphilic bacterial isolates. Data were analyzed by one-way ANOVA followed by Tukey post hoc test. Bars are Mean ± SD of three independent experiments. (**a**) The effect of pH on the growth of various isolates shows that SVUNM4 and SVUNM14 isolates respectively exhibited lowest and highest growths that were significantly different from other isolates at pH7 (^b,c,d,e,f^p < 0.05). At pH9, the isolates SVUNM9 and SVUNM13 respectively showed lowest and highest growths as compared to other isolates (^b,c,d,e,f^p < 0.05). While SVUNM9 isolate showed significantly highest growth as compared to other isolates, SVUNM14 showed lowest growth at pH10 (^b,c,d,e^p < 0.05). With the increase in pH further to 11 and 12, all isolates showed decreased growth trend as compared to that at lower pH. Among all isolates, the highest growth was exhibited by SVUNM14 and SVUNM4 at pH 11 and 12, respectively as compared to other isolates (^b,c,d,e^p < 0.05). (**b**) The effect of temperature on the growth of various isolates shows that SVUNM9 and SVUNM4 showed highest growth at temperatures 10 (^b^p < 0.05) and 25, respectively (^b,c,d^p < 0.05) compared to other isolates. At temperature 37, the lowest and highest growth were shown by SVUNM9 and SVUNM11, respectively (^a,b,d,e^p < 0.05) compared to other isolates. While the isolates SVUNM11 and SVUNM14 showed highest growth at temperature 44, SVUNM4 showed a significantly lower growth as compared to other isolates (^b,c,d,e^p < 0.05). Among all isolates, the lowest and highest growth were exhibited by SVUNM15 and SVUNM13, respectively at temperature 55 (^b,c,d,e^p < 0.05) compared to other isolates.

The growth-affecting parameters of the bacterial isolates were carried out at different alkaline pH values (7–11) (Fig. 1a) and temperatures (10, 25, 37, 44, and 55 °C) (Fig. 1b). The SVUNM4 and SVUNM14 isolates exhibited lowest and highest growth rates respectively that were significantly different from other isolates at pH7 (^b,c,d,e,f^p < 0.05). At pH9, the isolates SVUNM9 and SVUNM13 showed lowest and highest growth rates respectively as compared to other isolates (^b,c,d,e,f^p < 0.05). While SVUNM9 isolate showed significantly highest growth as compared to other isolates, SVUNM14 showed lowest growth at pH 10 (^b,c,d,e^p < 0.05). Further increase in pH to 11 and 12 decreased growth rates of all isolates as compared to that of pH 7 to 10. Among all isolates, SVUNM14 and SVUNM4 exhibited the highest growth at pH 11 and 12, respectively as compared to other isolates (^b,c,d,e^p < 0.05). The SVUNM9 and SVUNM4 showed highest growth at 10 °C (^b^p < 0.05) and 25 °C, respectively (^b,c,d^p < 0.05) compared to other isolates. At 37 °C, the SVUNM9 and SVUNM11 showed the lowest and highest growth respectively (^a,b,d,e^p < 0.05) compared to other isolates. While the isolates SVUNM11 and SVUNM14 showed the highest growth at 44 °C, SVUNM4 showed a significantly lower growth as compared to other isolates (^b,c,d,e^p < 0.05). Among all isolates, the SVUNM15 and SVUNM13 showed the lowest and highest growth respectively at 55 °C (^b,c,d,e^p < 0.05) compared to other isolates. The mean generation times of the isolates were calculated at room temperature and specific growth rates were calculated at different temperatures and pH by growing in their respective media. The mean generation time (td) ranged from 1 to 4.3 h, and the specific growth rate (u) ranged from 0.18 to 0.56 h amongst various isolates as specified in Table 2.

**Table 2:** Growth rate of the endospore forming bacterial isolates.

S. No	Name of the isolate	Specific growth rate (hours)
1	SVUNM4	39
2	SVUNM9	24
3	SVUNM11	21
4	SVUNM13	27
5	SVUNM14	21
6	SVUNM15	30

### Screening tests for the production of microbial calcite (CaCO_3_) precipitation

The ability of the bacterial isolates to produce CaCO_3_ was ascertained by performing the acid fizz, urease, and EDTA tests. All the strains were positive for these three tests.

### Microbial cementation in cement mortars

**Figure 2:**
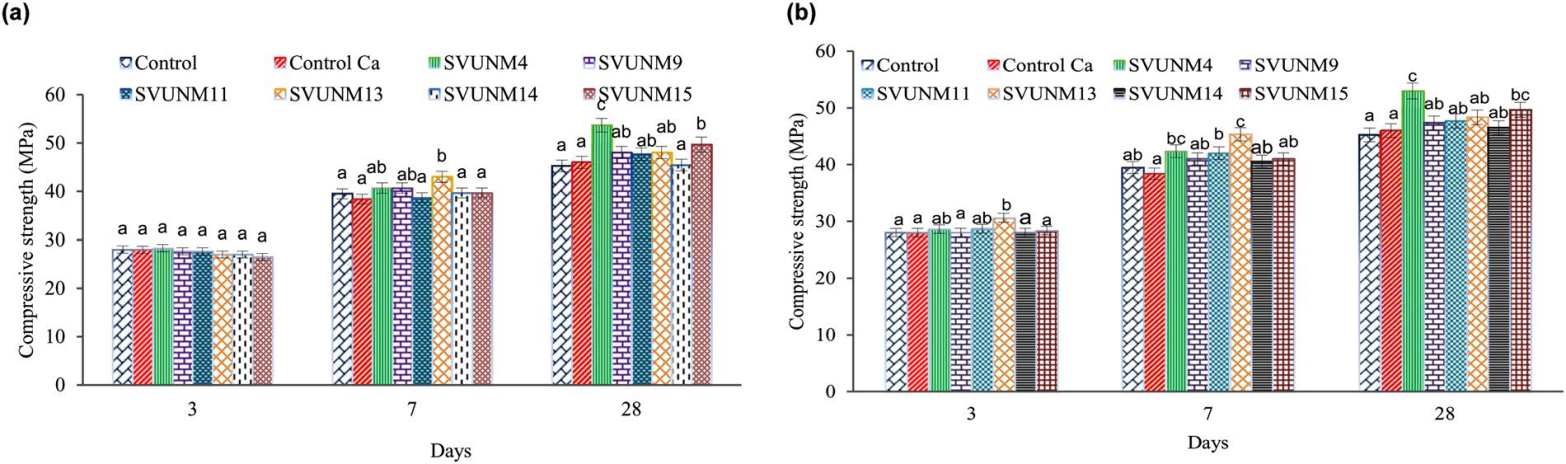
Effect of alkaliphilic isolates on comprehensive strength was compared across 8 isolates at 3 days, 7 days and 28 days. Data were analyzed by one-way ANOVA followed by Tukey post hoc test. Bars are Mean ± SD of three independent experiments. (**a**) Data clearly shows that there was no significant difference in comprehensive strength across various isolates (at 10^4^ cells/ml concentration) at 3 days (^a^p > 0.05). Whereas, the mean comprehensive strength value of SVUNM13 (at 10^4^ cells/ml concentration) at 7 days was found to be significantly higher (^b^p < 0.05) as compared to all other isolates except SVUNM4, and SVUNM9 (^ab^p > 0.05). Similarly, the mean comprehensive strength value of SVUNM15 at 28 days was found to be significantly higher (^b^p < 0.05) as compared to all other isolates except three isolates SVUNM9, SVUNM11, and SVUNM13 (^b^p < 0.05). We also noticed that the comprehensive strength value of SVUNM4 at 28 days was significantly higher (^c^p < 0.05) as compared to all other isolates. (**b**) When the effect of Alkaliphilic isolates (10^6^ cells/ml concentration) was compared, the mean comprehensive strength value of SVUNM13 at 3 days was found to be significantly higher (^b^p < 0.05) as compared to all other isolates (^a^p > 0.05) except two isolates (^b^p < 0.05); SVUNM4, and SVUNM11. At 7 days, the compressive strength of SVUNM11 (^b^p < 0.05) was found to be significantly higher and lower as compared to those of ‘controlled mortar with calcium lactate (^a^p > 0.05) and SVUNM13, respectively (^c^p < 0.05). Whereas, SVUNM13 showed a significantly higher compressive strength at 7 days (^c^p < 0.05) as compared to all other isolates except SVUNM4 (^b,c^p < 0.05). At 28 days, SVUNM4 showed highest compressive strength (^c^p < 0.05) as compared to all other isolates except SVUNM15 (^b,c^p < 0.05).

Mortar cubes were consolidated with CaCO_3_-forming bacteria at intervals of 7, 14 and 28 days to assess compressive strength. On the 3^rd^ day, the curing of all the specimens at different cell concentrations resulted in no significant improvement (^a^p > 0.05) in the strength of the consolidated mortar over the two controls (mortar with calcium lactate and mortar with broth). On 7^th^ day, the compressive strength test showed that the consolidated mortar cubes with *B. subtilis* SVUNM4 and *S. maltophilia* SVUNM13 isolates had significantly increased strength (^b^p < 0.05) over the controls, while the *B. methylotrophicus* SVUNM9, *P. dendritiformis* SVUNM11, *B. licheniformis* SVUNM14 and *Brevibacillus* SVUNM15 isolates showed less improvement. On the 28^th^ day, *B. subtilis* SVUNM4 (53.67 MPa) followed by *Brevibacillus* SVUNM15 (49.67 MPa) showed 28.63% and 22.1% increases in compressive strength at the 10^4^ cells/ml concentration when compared with the controls. *B. methylotrophicus* SVUNM9, *P. dendritiformis* SVUNM11, *S*. *maltophilia* SVUNM13, and *B. licheniformis* SVUNM14 showed slight improvements in compressive strength at 16%, 19.9%, 9.6% and 12.7%, respectively. Thus, *B. subtilis* SVUNM4 was identified as an appropriate sealing agent to improve the compressive strength of mortar cubes, demonstrating a 28.61% increase from the 3^rd^ day to the 28^th^ day (^c^p < 0.05) and a 9.6% increase in compressive strength on 28^th^ day over the controls, as shown in Fig. 2a.

On the 28^th^ day, compared with the controls, amongst the six tested isolates, *B. subtilis* SVUNM4 (53 MPa) followed by *Brevibacillus* SVUNM15 (49.66 MPa) showed 23.6% and 19.2% increases in compressive strength (^c^p < 0.05) at a concentration of 10^6^ cells/ml. *B. methylotrophicus* SVUNM9, *P. dendritiformis* SVUNM11, *S*. *maltophilia* SVUNM13 and *B. licheniformis* SVUNM14 showed slight improvements in compressive strength at 14%, 12.5%, 6.63% and 13.3%, respectively, as shown in Fig. 2b. However, on the 7^th^ day, SVUNM13 showed a significantly higher compressive strength (^c^p < 0.05) as compare to all other isolates.

Similarly, improvement in the cell concentration resulted in little decrease in compressive strength. Thus, the SVUNM4 isolate at a concentration of 10^4^ cells/ml is best and demonstrates that the alkaline nature of the ureolytic activity induces microbial CaCO_3_ precipitation. These results were produced using compressive strength test and scanning electron microscopy (SEM) studies of CaCO_3_ precipitation.

### Evaluation of crack repair in mortar cubes with simulated cracks at different cell concentrations

**Figure 3:**
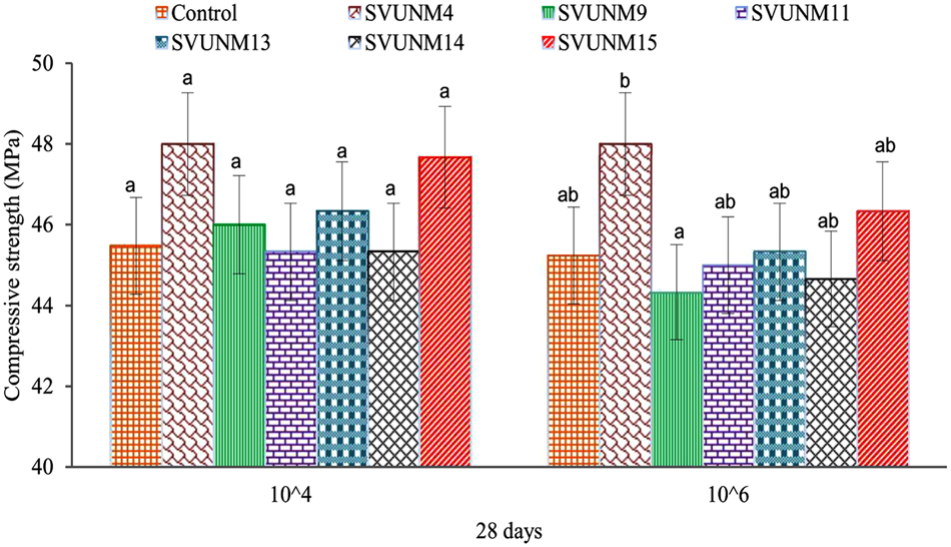
Effects of alkaliphiles on the self-healing capacity of mortar cubes cracked on the 7^th^ day. Data were analyzed by one-way ANOVA followed by Tukey post hoc test. Bars are Mean ± SD of three independent experiments. We noticed that there was no significant difference in comprehensive strength across various isolates at 28 days for 10^4^ cells/ml concentration (^a^p > 0.05). Whereas, the mean comprehensive strength value of SVUNM4 (^b^p < 0.05) at 28 days (10^6^ cells/ml concentration) was found to be higher as compared to all other isolates, but showed statistical significance only with SVUNM9 (^b^p < 0.05).

To assess the crack repairing abilities, bacterial isolates were tested at different cell concentrations (10^4^ and 10^6^ cells/ml) in a mortar cube by simulating cracks with a width of 1–3 mm and a depth of 10–50 mm. There was no significant difference in comprehensive strength across various isolates at 28 days for 10^4^ cells/ml concentration (^a^p > 0.05). Whereas, the mean comprehensive strength value of SVUNM4 at 28 days (10^6^ cells/ml concentration) was found to be higher (^b^p < 0.05) as compared to all other isolates, but showed statistical significance only with SVUNM9 (^b^p < 0.05; Fig. 3).

### Characterization studies using SEM

To evaluate microbial sand plugging in mortar cubes, all samples were examined under SEM for microbial CaCO_3_ precipitation (Fig. 4). Clear CaCO_3_ precipitation was observed in remediated crack areas. SEM indicated that CaCO_3_ crystals precipitated on the crack surface (Fig. 4b–g). The interior areas of the cracks showed the lowest CaCO_3_ precipitation, and bacterial presence was also scarce. CaCO_3_ crystals had distinct sharp edges, indicating a full growth of the crystals and it was observed to have rod-shaped holes, which were presumably the spaces occupied by bacteria in the most interior areas. These results indicate that microbial CaCO_3_ precipitation helps crack repair. The precipitation takes place due to reaction of isolates with moisture present in the air. As the crack width decreases from surface to inner periphery, the air contact is low and the crack width is less in the interior compared to the exterior surface of the concrete.

**Figuree 4:**
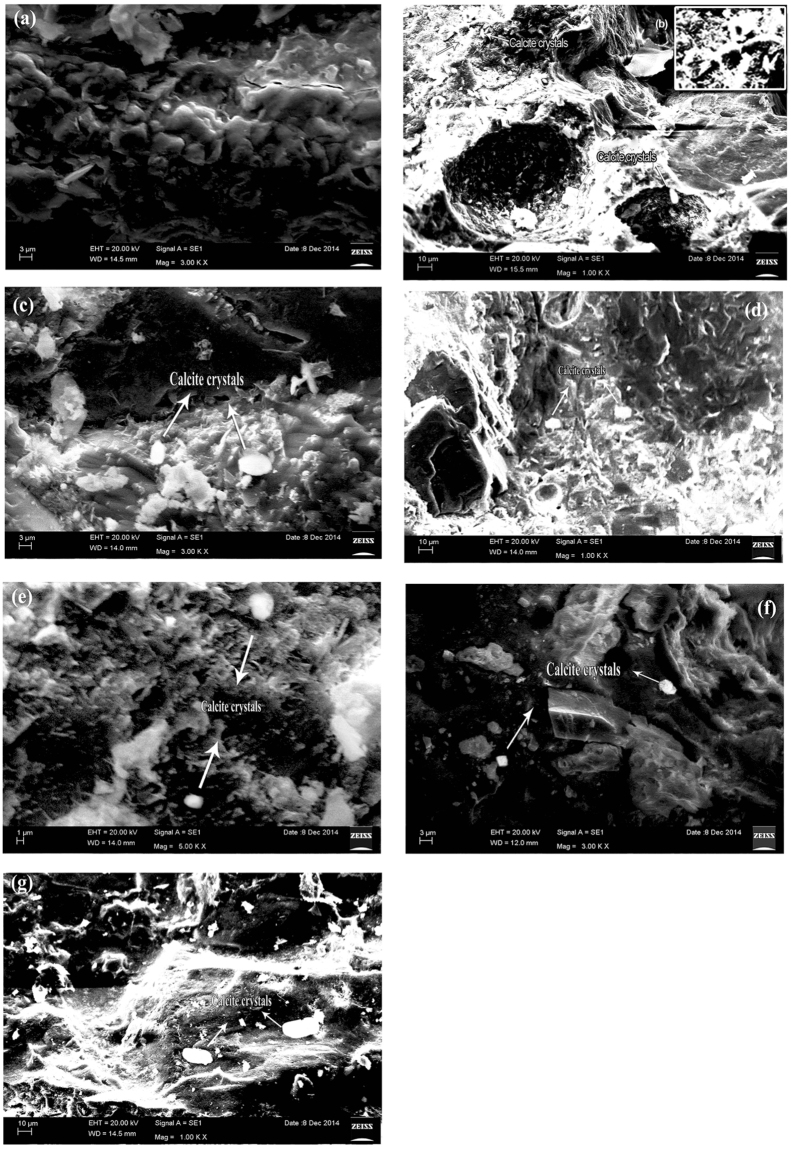
Scanning electron micrographs showing CaCO_3_ precipitation in mortar cracks. (**a**) Mortar was prepared without bacteria. Samples were taken from the nearest surface area at remediated cracks. Dense CaCO_3_ precipitation amidst an interior surface area of mortar cracks showing CaCO_3_ crystals with rod-shaped (**b**). *B. subtilis* SVUNM4 (**c**). *B. methylotrophicus* SVUNM9 (**d**). *P. dendritiformis* SVUNM11 (**e**). *S. maltophilia* SVUNM13 (**f**). *B. licheniformis* SVUNM14, and (**g**). *Brevibacillus* SVUNM15.

### Water impermeability

**Figure 5:**
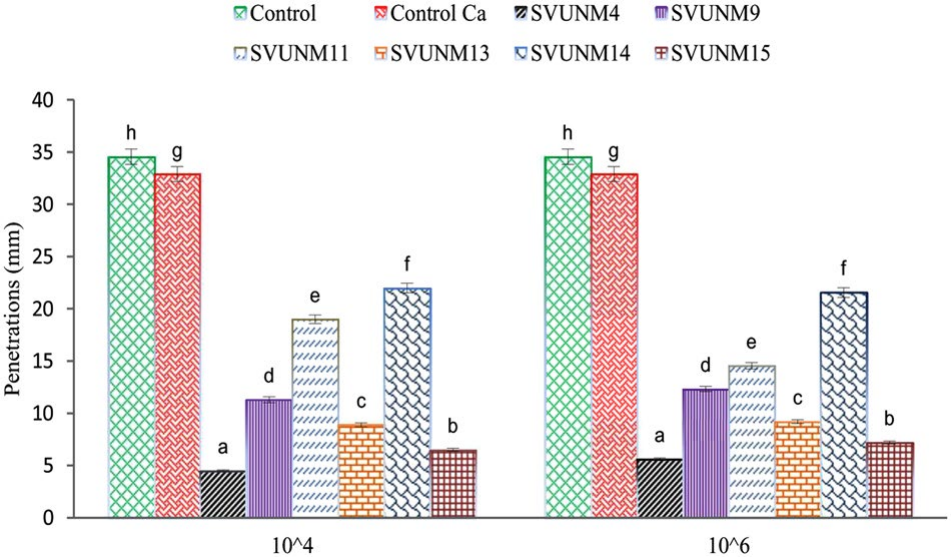
Effects of different alkaliphiles on the impermeability test. Data were analyzed by one-way ANOVA followed by Tukey post hoc test. Bars are Mean ± SD of three independent experiments. The isolates ‘controlled concrete (^h^)’ and SVUNM4 (^a^)’ respectively showed significantly highest and lowest penetration values as compared to other isolates (^b,c,d,e,f,g,h^p < 0.05) in either of the comparison groups 10^4^ or 10^6^ cells/ml concentration.

A concrete cube dimension of 150 mm^3^ was cast in water for control specimens. The test was performed with the DIN 1048 model. There was a large reduction in the depth of penetration of water in concrete cubes with all the bacterial isolates (Fig. 5) compared to that of the controls for each cell concentration. The average penetration was too low for the *B. subtilis* SVUNM4 isolate (^a^p < 0.05) when compared to other isolates at concentrations of 10^4^ cells/ml and 10^6^ cells/ml.

### Rapid chloride permeability test (RCPT) of concrete cubes with different cell concentrations after 60 days

Concrete was evaluated for chloride permeability as there is a potential risk of chloride-induced corrosion. In this test the concrete is subjected to electrical conductivity of a potential difference of 60 V, and the total charge passing through the sample at the end of 6 h is measured in Coulombs. The concrete cubes with SVUNM4 showed the lowest permeability, compared to other bacterial isolates (Tables 3, 4 and 5).

**Table 3:** Effect of various alkaliphiles on Rapid chloride permeability test (RCPT) at 10^4^ cells/ml.

S. No	TIME (Minutes)	CHARGE PASSED (Mille Ampere) 10^4^ cells/ml concentration						
		Control	SVUNM4	SVUNM9	SVUNM11	SVUNM13	SVUNM14	SVUNM15
1	0	68	39	33	32	32	43	38
2	30	72	32	34	36	36	44	35
3	60	74	36	36	38	38	47	33
4	90	78	34	37	42	42	52	38
5	120	85	31	39	44	44	54	32
6	150	79	36	40	43	43	55	39
7	180	84	37	42	45	45	59	30
8	210	86	38	38	46	46	62	35
9	240	88	32	44	48	48	65	38
10	270	82	34	41	44	44	67	36
11	300	84	41	40	45	45	70	33
12	330	79	34	39	42	42	63	38
13	360	75	39	41	43	43	58	36
I Cumulative		1925	848	934	1358	1021	1337	843
Charge in Coulombs		1733	763	841	1222	919	1239	763

**Table 4:** Effect of various alkaliphiles on Rapid chloride permeability test (RCPT) at 10^6^ cells/ml.

S. No	TIME (Minutes)	CHARGE PASSED (Mille Ampere) 10^6^ cells/ml concentration						
		Control	SVUNM4	SVUNM9	SVUNM11	SVUNM13	SVUNM14	SVUNM15
1	0	74	40	43	43	43	54	31
2	30	76	32	41	41	44	58	33
3	60	78	34	42	43	41	59	38
4	90	79	38	47	40	51	52	39
5	120	82	35	49	38	42	60	40
6	150	80	38	41	46	45	49	43
7	180	83	31	48	44	52	50	41
8	210	76	34	47	42	47	51	42
9	240	79	37	52	40	45	54	40
10	270	77	38	43	39	56	52	39
11	300	76	40	44	48	45	55	42
12	330	79	42	49	39	40	65	40
13	360	78	43	40	41	42	60	47
I Cumulative		1883	881	1089	1004	1101	1324	952
Charge in Coulombs		1695	793	980	904	991	1192	857

**Table 5:** Permeability class distribution based on RCPT report of different concrete specimens and evaluation of six alkaliphiles strains.

S. No	Treatment	Charge passed (Coulomb)		Permeability class as per ASTM1202
		10^4^ cells/ml concentration	10^6^ cells/ml concentration	
1	Control	1733	1695	MODERATE
2	SVUNM4	763	793	VERY LOW
3	SVUNM9	841	980	LOW
4	SVUNM11	1222	904	LOW
5	SVUNM13	919	991	LOW
6	SVUNM14	1239	1192	LOW
7	SVUNM15	763	857	LOW

## Discussion

Aerobic bacteria that have been isolated from deep horizons are the most efficient strains under environmental conditions that are very limited and in nutrient-desiccated environments. The mineral composition strongly influences the metabolic activities and colonization patterns of bacteria on minerals and rocks. According to a total chemical analysis, the mica used in this study contained different interlayer cations like 43.50% SiO_2_, 10.61% Al_2_O_3_, 2.83% Fe_2_O_3_, 4.66% FeO, 22.87% MgO, 0.773% CaO, 0.161% Na_2_O, 11.31% K_2_O, 0.034% MnO, and 0.19% TiO_2_ with a 1.48% loss of ignition and a total oxide summation of 98.42%^27^.

In this study, we isolated bacteria using simple nutrients because those bacteria that typically survive in oligotrophic environments prevail in subsurface environments due to extreme nutrient deprivation. Microbes may have cell walls and membranes that are very permeable to potential substrates, representing a considerable advantage. Indigenous microorganisms adapt to the conditions of subsurface environments. In subsurface microbiological studies, the aseptic procedure used for sampling is important. Auger drilling is not suitable for coring sediments more than 30 mts in depth. Therefore, in our study, we used an air drilling method to collect samples from the roof and walls. In contrast to our prior studies^28,29^, we used a combination of mud-rotary drilling and split spoon coring to obtain sediments at a depth of 2000 mts. The mud-rotary drilling technique is a complex process initiated when cells sense the presence of adverse conditions such as the lack of substrates, excessive heat or dryness or osmotic stress^23^. *Bacillus* is widely distributed in soils and sediments and is capable of both fermentative and respirative metabolism. Members of the genus *Bacillus* have been isolated from a shallow aerobic ground water system^30^. Similarly, in our study, we identified spores as members of the genera *Bacilli* and *Stenotrophomonas*. This result suggests that spore-forming bacteria may be common in deep subsurface environments.

The geochemical activities of microorganisms, especially bacteria are responsible to a great extent for the deposition of minerals throughout the Earth’s history. These microorganisms induce or control the precipitation of different minerals, such as oxides, sulphates, phosphates, and carbonates^31^. Calcium carbonate has been proved to be the most suitable fillers in concrete due to its high compatibility with cementitious compositions and is precipitated through a microbiologically induced mineralization process in the presence of a calcium source. So it is used in concrete construction materials because cracks decrease the lifespan of concrete structure. In addition, crack repair is more expensive due to the cost of the concrete life cycle and labour during repair^32^.

The microbial CaCO_3_ precipitation process is useful for the remediation of the surface and subsurface of porous media. The process of microbial mineral plugging in porous media was induced by using an alkhalophilic soil microorganism, *B. pasteurii*. Urease activity is high at an alkaline pH, at which CaCO_3_ precipitation is favourable^33^. Figure 4 shows scanning electron micrographs of CaCO_3_ precipitation in mortar cracks. Though all the samples showed calcite crystal precipitation, only SVUNM4 showed increased compressive strength because the bacteria in SVUNM4 is capable of producing more calcite crystal bonds than other bacteria used in this study. Mortar cubes were prepared without bacteria as shown in Fig. 4a. As shown in Fig. 4b–g, the sample surface areas of remediated cracks showed dense CaCO_3_ precipitation between the interior surface areas of mortar cracks and CaCO_3_ crystals with rod-shaped *B. subtilis* SVUNM4, *B. methylotrophicus* SVUNM9, *P. dendritiformis* SVUNM11, *S. maltophilia* SVUNM13, *B. licheniformis* SVUNM14, and *Brevibacillus* SVUNM15. Large amounts of precipitation were evident at crack surfaces, and apparent CaCO_3_ polymorphs were observed on the surfaces of bacteria-based specimens, suggesting that their formation is linked to bacterial activity^34,35^. The capacity of *B. pasteurii* to fill cracks was observed by using artificially cracked cement mortar beams. In this study, *B. pasteurii* was used as a remediation for cracks and fissures through microbiologically induced CaCO_3_ precipitation in concrete^21^. It was reported that bio-deposition improved the durability of cement mortar/concrete specimens. Additionally, the deposition of CaCO_3_ crystals decreased water absorption to lock the porosity of specimens, leading to a decrease in the carbonation rate of approximately 25–30%.

As it is essential to improve the strength and durability of concrete, similar biomimetic techniques have been used to improve the performance of the concrete structures. However, no improvement in the strength of cement mortar cubes was achieved using *Escherichia coli*^22^. In this study the compressive strength of all bacterial isolates was observed at different cell concentrations. As shown in Fig. 2(a,b), the compressive strength of different cell concentrations increased in mortar cubes that contained microbial cells irrespective of the media compared with two controls on the 3^rd^, 7^th^ and 28^th^ days. When the compressive strength was compared, *B. subtilis* SVUNM4 and *Brevibacillus* SVUNM15 had the highest strengths at a cell concentration of 10^4^. A biological repair technique using ureolytic bacteria, such as *B. sphaericus*, was previously shown to precipitate CaCO_3_ via the conversion of urea into ammonium and carbonate^36^. Immobilized ureolytic and denitrifying bacteria reportedly affect the compressive strength of protective materials. This study showed that the application of *B. sphaericus* decreased the compressive strength of concrete cubes on the 7^th^ and 28^th^ days by 63% and 60%, respectively. Similarly the utilization of denitrifying bacteria, *Diaphorobacter nitroreducens* caused a reduction in the compressive strength on both the 7^th^ and 28^th^ days of curing^37^.

The effects of ASTM C1202-05 (ASTM, 2005) RCPT on cylindrical concrete samples at concentrations of 10^4^ and 10^6^ cells/ml are shown in Tables 3, 4, and 5; these are the average outputs of the RCPT analysis. The permeability of control concrete specimens was moderate, while specimens SVUNM9, SVUNM11, SVUNM14 and SVUNM15 treated with concentrations of 10^4^ and 10^6^ bacterial cells/ml were observed to be low, and the SVUNM4 resulted in a very low permeability as shown in Table 5. The average charges for control samples were 1733 and 1695 Coulombs, whereas samples prepared with different bacterial cells were 763, 763, 841, 919, 1222 and 1239 Coulombs for the 10^4^ cell concentration and 793, 980, 904, 991, 857 and 1197 Coulombs for the 10^6^ concentration as shown in Tables 3 and 4. Consequently, bacterial involvement considerably decreased the chloride permeability of the concrete.

Adding *B. pasteurii* (at 10^5^ cells/ml) to mortar specimens increased strength and reduced both water absorption and chloride penetration^38^. The difference in concrete strength depends on the form of the added bacteria (0.5% w/w cement)^37^. When bacterial spores were added, foaming of the proteins and the corresponding increase in porosity reduced the ultimate strength of the concrete by 60%, while vegetative cells were discovered to improve the self healing capacity of the concrete^39^. The positive potential of biomineralized CaCO_3_ is useful for the repair of concrete cracks by *B. sphaericus*^40^. Furthermore, the compressive strength of biomineralized specimens might be re-established to 84%^41^. *B. pseudofirmus* has emerged as a fitting strain to act as a self-healing moderator for bio-mineralization in cement-based resources based on sporulation, germination, *in vitro* calcium carbonate precipitation under alkaline conditions and the capacity of spores to survive within concrete^42^. The microbiological CaCO_3_ precipitation of concrete has been examined carefully for its capacity to enhance the compressive strength of concrete. Four CaCO_3_-forming bacteria (CFB) were studied from seven environmental concrete structures, and bacterial species related to *Arthrobacter crystallopoietes* enhanced the compressive strength of concrete cubes^43^.

The CaCO_3_-precipitating effects of the bacterium, *Bacillus subtilis* showed a 30% increase in the compressive strength of bacterial shotcrete specimens compared to control specimens, thus improving the mechanical properties of shotcrete. The presence of bacteria in the mix design and curing solution enhances the tensile strength, and decreases the water absorption and porosity of shotcrete^44^. The incorporation of vegetative bacterial cells increased compressive strength of mortar specimens compared to control specimens^45^. The application of the halophilic bacterium, *Exiguobacterium mexicanum* isolated from sea water, showed a 23.5% increase in compressive strength and a 5-fold reduction in water absorption of concrete specimens under 5% salt stress conditions^46^. The non-ureolytic bacteria, *Bacillus cohnii* was reported to increase the compressive strength by 49%. *Bacillus* sp. CT-5-treated reinforced concrete (RC) specimens reduced the corrosion rate and mass, and increased the pull-out strength^47^. Another bacterial strain*, B. subtilis* showed 28% increase in the compressive strength the bacteria-incorporated concrete compared to that of control concrete with an optimum concentration of 10^6^ cells/ml^48^. Similarly, in our study, the isolates showed a high-quality increase in compressive strength compared to the controlled concrete without bacteria and calcium lactate. The volume of bacterial culture is about 85 liters per m^3^ of concrete to maintain water to microorganism ratio of 0.47. The total liquid (water + microorganism) is about 160 liters maintaining a liquid/cement ratio of 0.5, which produces M20 grade concrete according to IS 456 code of practice. About 320 kgs of cement along with 960 kgs of sand have to be used to maintain a ratio of 1:3. The above ratios could be used in bio concreting works by producing the required quantity of bacteria and mixing it with cement and sand along with water for eco-friendly sustainable concrete and underground concrete structures.

## Conclusions

Compressive strength studies using mortar cubes were carried out by adding the alkaliphilic strains *B. subtilis* SVUNM4, *B. methylotrophicus* SVUNM9, *P. dendritiformis* SVUNM11, *S. maltophilia* SVUNM13, *B. licheniformis* SVUNM14, and *Brevibacillus* SVUNM15 at concentrations of 10^4^ and 10^6^ cells/ml along with control mix. According to the strength studies, the compression strength of *B. subtilis* SVUNM4 with a concentration of 10^6^ increased by 23.6% over other isolates. For all the strains, strength improvements were significantly higher (28.61%) with a concentration of 10^4^ cells/ml. We also studied the self-healing capacity of the isolates in simulated crack mortar cubes. Self-healing capacity was measured using a compressive strength test and performing SEM analysis of CaCO_3_ precipitation. *B. subtilis*, SVUNM4 prevented chloride penetration in a concrete structure and showed the highest self-healing ability, whereas SVUNM14 showed the lowest self-healing capacity compared to the rest of the isolates. In the present study, we found that, compared to the other isolates, *B. subtilis* SVUNM4 demonstrated the highest strength and effective self-healing capacity. Our research is critical for enhancing the development of eco-friendly self-healing bio-concrete.

## Materials and Methods

### Sampling site

In the current study, we chose an ancient subsurface mica mine that was dug up to approximately 100 mts in depth and is located in Gudur division, Nellore district, Andhra Pradesh, India, at a latitude of 14°10′ and 14°19′, longitude 79°35′ and 79°45′.

### Collection and processing of pegmatite mica

Ore samples were collected from various operating pegmatite mica mines hanging on a wall and footwall contacts using sterile cable tool drilling. Soon after collection, the mica samples were pooled in a freezer at the drilling site. The milled mica samples of pH 9 to 11 were cut into 0.2-mm mesh and used for the isolation of endospore-forming bacteria.

### Isolation of endospore-forming bacilli

The aerobic endospore-forming bacteria in the pegmatite samples were isolated by way of a serial dilution plate technique. We added 10.0 g of mica samples to 90 ml of sterile 0.5% saline solution, vigorously mixed by vortexing and incubated the samples at 80 °C for 30 min to selectively isolate spores. The samples were serially diluted up to 10^5^, and 100-μl suspensions were spread on nutrient agar (NA) plates including methyl red (0.02%). The plates were incubated at 37 °C for 48 h. Colonies with distinct cultural characteristics were picked randomly and purified by repeated streaking on NA medium. The isolates were maintained on slants at 4 °C until further use.

### Molecular identification of the isolates

Genomic DNA was isolated from the 50 ml of liquid bacterial culture using a Genei ultrapure bacterial genomic purification kit (KT-162) (Genei Private Limited, Bangalore) according to the manufacturer’s instructions and stored at 20 °C until further use. Amplification of the 16S ribosomal DNA of endospore-forming isolates was carried out using the primers F8 (5′ GAGTTTGATCATGGCTCAG 3′) and R1407 (5′ TTCAGCATTGTTCCATTGGCA 3′) by polymerase chain reaction (PCR). The reaction mixture was heated for 2 minutes at 96 °C, and then amplification was carried out in a master cycler (Eppendorf, Germany) consisting of an initial denaturation step at 94 °C for 5 min, then 35 cycles of 94 °C for 30 s, 55 °C for 30 s, 72 °C for 1.30 min and a final extension at 72 °C for 10 min. The PCR products were resolved by 0.8% agarose gel along with a Stepup 500 bp DNA ladder (Genei Private Limited, Bangalore). Then, the PCR products were sequenced bi-directionally using forward, reverse and internal primers (27 f, 515 f and 1492 f)^49^ with ABI prism 377 automatic sequencers (Applied Biosystems, Foster City, CA, USA). The sequence data obtained from the isolates were aligned with 16S rRNA sequences in the GenBank database using the BLAST programme to identify the closely related homologies of the isolate^50^.

### Morphological and biochemical characterization

The isolates were characterized based on their morphological features according to Gram staining, motility, shape, and others. To biochemically characterize the above isolates, various tests such as the oxidation-fermentation test, catalase test, indole test, methyl red test, Voges-Proskauer test, citrate utilization test, starch hydrolysis, gelatin hydrolysis, sugar fermentation, nitrate reduction test and hydrogen sulphide production test were performed to identify the production of acid or gas from glucose, sucrose and mannitol^51^.

### Growth studies

Basic optimization of growth-influencing parameters was performed at different pH values (7, 9, 10, 11, 12) and temperatures (10, 25, 37, 44, 55 °C). The mean generation time (td) and specific growth rate (μ) were determined by increasing isolates in the appropriate media at optimum pH and temperature. The growth rate was observed by measuring the absorbance at 650 nm for a standard period of time. Time (h) was plotted against optical density, and the td was obtained as described below:

$${\rm{td}}=0.693/{\rm{\mu }}$$

### Screening tests to detect the production of microbial-induced CaCO_3_ precipitation by urease

Urease activity was detected according to Christensen^52^.

### Acid fizz test

Overnight cultures of bacteria were inoculated individually in mineral salt medium (MSM) with 5% calcium lactate pentahydrate. Bacteria were maintained as controls without calcium lactate and were incubated for 3 days to assess the utilization of calcium lactate as limestone. After 3 days, the supernatants were collected by centrifugation at 16,800 rpm × 20 min at room temperature and observed for effervescence in the form of gas bubbles by adding two drops of 10% HCl.

### EDTA test

An EDTA test was performed to estimate calcium ions using EDTA titration according to Mcleon^53^.

### Microbial cementation in cement mortars

All endospore-forming, urease (+Ve), acid fizz (+Ve), and EDTA (+Ve) CaCO_3_-forming bacteria were incubated in nutrient broth containing calcium lactate, and cell cultures were incubated in nutrient broth containing calcium lactate at 30 °C for 24 h. Cell cultures were centrifuged at 8000 rpm and washed twice with 50 mM sodium phosphate buffer (pH 7.5). Bacterial suspensions of 10^4^ and 10^6^ cells/ml were subsequently created using 50 mM sodium phosphate buffer and adjusted to 0.6 at OD 600 nm. A mixture of cement and sand was created at a ratio of 1:3 (by weight), and water and cement were mixed at a ratio of 0.50. The sand and cement were thoroughly mixed, and bacterial culture broth was added corresponding to the OD at 600 1.0. The water and bacterial culture ratio was maintained at 0.47. A cube mould (70.6 mm × 70.6 mm × 70.6 mm) was cast as per IS 4031 (part 6) 2000 and compacted in a vibration machine. Demoulded cubes were cured at room temperature until performing compression tests at 3, 7, and 28 days. All samples were prepared in approximately 9 sets. Calcium lactate and deionized water without bacteria were used as the controls. After removing from water, each concrete cube was completely air dried at 25 °C prior to a compression test. The compressive strengths of the specimens were measured with an automatic compression testing machine (COMPTEST)^54^.

### Evaluation of crack repair in mortar cubes with simulated cracks at different cell concentrations

A cube mould with the dimensions 70.6 mm × 70.6 mm × 70.6 mm was prepared as described previously. The cubes were cut to maintain an average width of 1 mm to 3 mm and a depth of 10 mm to 50 mm to simulate cracking. The cracks in the cubes were remediated with efficient bacterial cells combined with natural sieved sand. Microbial plugging of the mortar cracks was examined using different concentrations of cells (10^4^ and 10^6^ cells/ml) mixed with sand. The cracks in the control specimens were filled with natural sieved sand and water. Both sets of cubes were cured in water for 28 days. Compression testing was performed using the COMPTEST 3000 machine.

### Characterization studies using SEM

CaCO_3_ formation via biomineration was analysed using direct and microscopic observations. To conduct SEM analysis, broken mortar/concrete test samples were maintained in a desiccator overnight to remove moisture. Then, all the test samples were layered with gold and were analysed in a zesis EV050 scanning electron microscope at 20 kv.

### Quality control of cement concrete cylinders

To ensure the strength and durability of the cement concrete cylinders, a water permeability test and an RCPT were performed.

### Impermeability of concrete cubes after 28 days

For the water impermeability test, concrete cubes with the dimensions 150 mm × 150 mm × 150 mm (M20 grade) were prepared with cultures at different concentrations (10^4^ and 10^6^ cells/ml). Ordinary Portland cement, fine aggregate (medium-sized natural/river sand) and crushed stone coarse aggregate with a maximum size of 20 mm were used in the concrete. The ratio of cement, sand, and coarse aggregate was 1:1.54:2.86. The water/bacterial culture–cement ratio was maintained at 0.47. Control samples (without bacterial cells) were also prepared. After air drying, the specimens were firmly secured in a position in the test apparatus (AIMIL India Ltd, Delhi) and analysed in a water impermeability test as per German standard DIN 1048. Water nozzles with adjustable pressure were connected to the surface of each cube. The bottom side gasket was thoroughly checked to avoid water leakage when the sample was subjected to water and atmospheric pressures of 1, 3 and 7 bars applied sequentially for 24 h. When the samples were subjected to 7 kg/cm^2^ pressure, we checked whether any water leakage occurred; if there was any drop in the pressure, it was adjusted to 7 kg/cm^2^ immediately. The penetration depth of water into the concrete was measured and marked with paint^55^.

### RCPT of concrete cubes after 60 days

The effects of different concentrations (10^4^ and 10^6^ cells/ml) of efficient bacteria were also studied to determine the optimum load to attain maximum compressive strength as described for the control strain. A cube mould of 150 mm^2^ was prepared as described above. The above-described cubes were provided with a cut to simulate cracks. The width of the cut was maintained at an average of 3 mm (0.118 in), and the depth was 13.4 mm. The cracks in the cubes were remediated after 21 days. These remediated mortar cubes were also subjected to a compression test using an automatic compression testing machine (COMPTEST 3000)^56^.

### Statistical analysis

All the experiments were carried out in triplicate and expressed as mean ± SD. One-way ANOVA was used to test the significance of differences across different isolates followed by Tukey- Post Hoc Method. Statistical analysis was performed using SPSS (version 20; IBM SPSS Inc., Chicago, 1 L, USA).

## Electronic supplementary material

SUPPLEMENTARY INFORMATION
