# Supplementary material for: Subsurface Endospore-Forming Bacteria Possess Bio-Sealant Properties
Source: Sci Rep. 2018 Apr 24;8:6448. doi: 10.1038/s41598-018-24730-3 (PMC5915551; doi:10.1038/s41598-018-24730-3)
Supplement: Supplementary file 1 — SUPPLEMENTARY INFORMATION [file 41598_2018_24730_MOESM1_ESM.doc]

**SUPPLEMENTARY INFORMATION**

**Subsurface endospore-forming bacteria possess bio-sealant properties**

Basha Sreenivasulu^1*^, Lakshman Kumar Lingamgunta^2^, Jayakumar Kannali^1^, Swarna Kumari Gajula^1^, Ramesh Bandikari^2,3^, Sreenivasulu Dasari^2^, Veena Dalavai^1^, Paramageetham Chinthala^1^, Prasada Babu Gundala^1^, Peera Kutagolla^4^, Vinodh kumar Balaji^5^

^1^Department of Microbiology, Sri Venkateswara University, Tirupati-517502, Andhra Pradesh, India

^2^Department of Biochemistry, Sri Venkateswara University, Tirupati-517502, Andhra Pradesh, India

^3^College of Life Science and Technology, State key Laboratory of Agricultural Microbiology, Huazhong Agricultural University, Wuhan 430070, China

^4^Department of Zoology, Sri Venkateswara University, Tirupati-517502, Andhra Pradesh, India

^5^Department of Civil Engineering, Siddharth Institute of Engineering & Technology, Puttur-517583, India

**^*^Corresponding author:** Dr. Basha Sreenivasulu**,** Email: [bashasreenivasulu@gmail.com](mailto:bashasreenivasulu@gmail.com)

**Table-1S. Distance Matrix based on Nucleotide Sequence Homology Using Kimura-2 Parameter for isolate SVUNM4***

* Sample M4, can be referred as SVUNM4

Accession No : Name of the isolate

JF 411055 : *Bacillus* sp.17 (2011)

GQ375226 : *Bacillus subtilis*, subsp. *Subtilis* strain CICC 10020

AB440270 : *Bacillus subtilis gene*

AB440269 : *Bacillus subtilis gene*

EU221334 : *Bacillus subtilis strain* JM1C6

DQ993674 : *Bacillus subtilis strain BCRC10058*

AB210982 : *Bacillus subtilis strain:* SSCT51

DQ990020 : *Bacterium* 1-Gw2-2

EU047884 : *Bacillus subtilis strain BS3902*

GU191916 : *Bacillus subtilis subsp. Subtilis stain* SB3130

**Table-2S. Distance Matrix based on Nucleotide Sequence Homology Using Kimura 2 Parameter for isolate SVUNM9***

* Sample M9, can be referred as SVUNM9

Accession No : Name of the Bacteria

JN086147 : *Bacillus* *amyloliquifaciens* st. RX-35

JN086146 : *Bacillus* *amyloliquifaciens* st. RX-34

JF899287 : *Bacillus* *methyltrophicus* Ht 10-2

JN086143 : *Bacillus* *amyloliquifaciens* st. DX-18

HM055608 : *Bacillus* *amyloliquifaciens* st. – 3S

GU323369 : *Bacillus* *amyloliquifaciens* st. HS8

IF460743 : *Bacillus* *methyltrophicus* st. KS8-18

HQ831404 : *Bacillus* *methyltrophicus* st. NS1-29

HQ433576 : *Bacillus* sp. 19(2010)

HQ662601 : *Bacillus* *methyltrophicus* st. MO-BM-16

**Table-3S. Distance Matrix based on Nucleotide Sequence Homology Using Kimura-2 Parameter for isolate SVUNM11***

* Sample M11, can be referred as SVUNM11

Accession No : Name of the Bacteria

NR042861 : *Paenibacillus* *dendritiformis s*train C1P-105967

HM071942 : *Paenibacillus* *dendritiformis* strain P4-11

HQ625389 : *Paenibacillus* *dendritiformis* strain RRLKE4

HQ330529 : *Paenibacillus* sp. AT2

Y16129 : *Paenibacillus* sp. C-168

EU330645 : *Paenibacillus* *thiaminolyticus* 8118

JN215506 : *Paenibacillus thiaminolyticus* strain NB12

AB680934 : *Paenibacillus* *thiaminolyticus* gene strain NBRC15656

AJ320490 : *Paenibacillus thiaminolyticus* strain DSM 72627

JF309262 : *Paenibacillus thiaminolyticus* sp 3504BRRJ

**Table-4S. Distance Matrix based on Nucleotide Sequence Homology Using Kimura-2 Parameter for isolate SVUNM13***

* Sample M13, SVUNM13

Accession No : Name of the isolate

JN 644502 : *Stenotrophomons maltophilia* strain

EF509820 : Uncultured bacterium clone P4D7-617

JN400504 : *Xanthomonas sp* P4LE-116S ribosomal RNA gene

JN230476 : Uncultured bacterium clone 3H^3^C 13

FN645733 : *Xanthomonas sp.* KD2009-18 16S rRNA

AB617535 : *Pseudomonas s*p. MSK534 gene

GU372745 : *Stenotrophomonas* sp*.* strain FE 152

GQ 416607 : Uncultured *Stenotrophomonas sp.* clone F70

DQ 256392 : *Stenotrophomonas* sp LQX11

JQ359085 : *Stenotrophomonas* sp. DOB6

**Table-5S. Distance Matrix based on Nucleotide Sequence Homology Using Kimura-2 Parameter for isolate SVUNM14***

* Sample M14, can be referred as SVUNM14

Accession No : Name of the Isolate

FR 823409 : *Bacillus* sp. LtCr40 isolate LTCr40

FN666245 : *Bacillus licheniformis* Strain AE6

AB525389 : *Bacillus licheniformis* gene

AM 913927 : *Bacillus* sp L157 Isolate L157

EF471917 : *Bacillus* sp. J24

JN792201 : *Bacillus* sp. KKCVCW1

DQ 416780 : *Bacillus* sp. GIDM-7

EU362149 : *Bacillus* sp. B3 120081

JF772468 : *Bacillus* sp. BD3 (2011)

EU445292 : *Bacillus licheniformis* strain RPK

**Table 6S. Distance Matrix based on Nucleotide Sequence Homology Using Kimura-2 Parameter for isolate SVUNM15***

***** Sample M15, can be referred as SVUNM15

Accession No : Name of the Isolate

GU 205165 : *Bacterium* HWB3

AB680273 : *Brevibacillus parabravis* gene

HQ231211 : *Brevibacillus s*p. N3

AB2151A1 : *Brevi bacillus parabrevis* gene

AB 680065 : *Brevi bacillus parabrevis* gene NBRC12374

EF990684 : *Brevi bacillus* sp. B2 (2007)

EF 582417 : *Brevi bacillus parabrevis* strain HDYM-19

JN315628 : *Brevi bacillus parabrevis* strain ABRJJ2

HQ 231210 : *Brevi bacillus* Sp. N­­_2_

**Fig. 1S. Phylogenetic tree constructed using Neighbour Joining method for isolate SVUNM4***

* Sample M4, can be referred as SVUNM4

**Fig. 2S. Phylogenetic Tree Constructed using Neighbour Joining method for isolate SVUNM9***

***** Sample M9, can be referred as SVUNM9

**Fig. 3S. Phylogenetic Tree Constructed using Neighbour Joining method for isolate SVUNM11***

***** Sample M11, can be referred as SVUNM11

**Fig 4S. Phylogenetic Tree Constructed using Neighbour Joining method for isolate SVUNM13***

***** Sample M13, can be referred as SVUNM13

**Fig. 5S. Phylogenetic Tree Constructed using Neighbour Joining method for isolate SVUNM14***

***** Sample M14, can be referred as SVUNM14

**Fig. 6S. Phylogenetic Tree Constructed using Neighbour Joining method for isolate SVUNM15***

***** Sample M15, can be referred as SVUNM15
